# Assessment of FSDAF Accuracy on Cotton Yield Estimation Using Different MODIS Products and Landsat Based on the Mixed Degree Index with Different Surroundings

**DOI:** 10.3390/s21155184

**Published:** 2021-07-30

**Authors:** Linghua Meng, Huanjun Liu, Susan L. Ustin, Xinle Zhang

**Affiliations:** 1 Northeast Institute of Geography and Agroecology, Chinese Academy of Sciences, Changchun 130102, China; menglinghua@iga.ac.cn; 2 University of Chinese Academy of Sciences, Beijing 100049, China; 3 Center for Spatial Technologies and Remote Sensing (CSTARS), Department of Land, Air, and Water Resources, University of California, Davis, CA 95616, USA; slustin@ucdavis.edu; 4 School of Information Technology, Jilin Agricultural University, Changchun 130102, China; zhangxinle@gmail.com

**Keywords:** FSDAF, mixed pixel, cotton growth, field scale, MDI

## Abstract

Research on fusion modeling of high spatial and temporal resolution images typically uses MODIS products at 500 m and 250 m resolution with Landsat images at 30 m, but the effect on results of the date of reference images and the ‘mixed pixels’ nature of moderate-resolution imaging spectroradiometer (MODIS) images are not often considered. In this study, we evaluated those effects using the flexible spatiotemporal data fusion model (FSDAF) to generate fusion images with both high spatial resolution and frequent coverage over three cotton field plots in the San Joaquin Valley of California, USA. Landsat images of different dates (day-of-year (DOY) 174, 206, and 254, representing early, middle, and end stages of the growing season, respectively) were used as reference images in fusion with two MODIS products (MOD09GA and MOD13Q1) to produce new time-series fusion images with improved temporal sampling over that provided by Landsat alone. The impact on the accuracy of yield estimation of the different Landsat reference dates, as well as the degree of mixing of the two MODIS products, were evaluated. A mixed degree index (MDI) was constructed to evaluate the accuracy and time-series fusion results of the different cotton plots, after which the different yield estimation models were compared. The results show the following: (1) there is a strong correlation (above 0.6) between cotton yield and both the Normalized Difference Vegetation Index (NDVI) from Landsat (NDVI_L30_) and NDVI from the fusion of Landsat with MOD13Q1 (NDVI_F250_). (2) Use of a mid-season Landsat image as reference for the fusion of MODIS imagery provides a better yield estimation, 14.73% and 17.26% higher than reference images from early or late in the season, respectively. (3) The accuracy of the yield estimation model of the three plots is different and relates to the MDI of the plots and the types of surrounding crops. These results can be used as a reference for data fusion for vegetation monitoring using remote sensing at the field scale.

## 1. Introduction

Time-series data from satellite images with frequent coverage are important for studying land surface dynamics, such as monitoring vegetation phenology [1,2], detecting land cover and land-use change [3], and estimating agriculture intensity [4]. Additionally, accurate spatiotemporal information about the crop condition during the growing season is critical for crop management and yield estimation [5,6,7]. It is important to be able to estimate crop yields before harvest for food security and commodity trading purposes.

Satellite images with a long return cycle with fine spatial resolution and a short return cycle with coarse resolution can be used for applications that require imagery from the past several decades. Data from the moderate resolution imaging spectroradiometer (MODIS) and the National Oceanic and Atmospheric Association (NOAA) advanced very high-resolution radiometer (AVHRR) have coverage every 1–2 days, but the spatial resolution is coarse at 250 m to 1 km. The other type of data have a fine spatial resolution, but the revisit cycle is long. It is difficult to acquire remote sensing (RS) images with both high spatial resolution and frequent coverage due to technical and budget limitations [8]. For example, RS images acquired from the Landsat and SPOT series and Indian remote sensing (IRS) satellites with spatial resolutions from 6 to 30 m are usually the primary data source for land use/land cover mapping and change detection [9], ecosystem dynamic monitoring [10,11] and biogeochemical parameter estimation [12]. However, these satellites have longer return cycles (e.g., Landsat is 16 d; IRS is 24 d) and are subject to frequent cloud contamination and other poor atmospheric conditions [13]. These conditions have limited the application of these satellites in detecting rapid surface changes associated with intra-seasonal ecosystem variations and natural disasters [14]. In contrast, MODIS has a shorter revisit cycle and can provide frequent observations, but the spatial resolution is coarse [15]. As such, MODIS data cannot meet the requirements for surface cover change and ecosystem monitoring at farm and field scales. Thus, the fusion of data from different types of sensors has become a feasible and less costly method to improve the utility of RS data to monitor surface dynamics at the local level [16].

Spatiotemporal data fusion methods of high spatial and temporal resolution images have been developed to blend these two types of satellite images to generate synthesized data with both high spatial resolution and frequent coverage. Among the weighted function-based methods, the spatial and temporal adaptive reflectance fusion model (STARFM) was the first developed [17]. STARFM assumes that changes in reflectance are consistent and comparable at MODIS and Landsat thematic mapper (TM) resolutions and therefore predicts pixel values using a function that gives a higher weight to more pure MODIS pixels based on information from neighboring Landsat TM pixels. Although STARFM is easy to understand and widely used, it still has the following problems: (1) STARFM had a good effect in predicting the gradient information, but it cannot predict the instantaneous disturbance events in the short term. (2) STARFM did not consider reflectance directionality. (3) STARFM assumed that MODIS pixel is pure and homogeneous. In response to these problems, scholars have made improvements; the spatial–temporal adaptive algorithm for mapping reflectance change (STAARCH) and the enhanced STARFM method (ESTARFM) were two other examples of trials to improve the original STARFM to detect the land-cover change and make it applicable in the heterogeneous landscape [18,19].

Emelyanova et al. conducted a comprehensive study to investigate the performance of STARFM and ESTARFM in two landscapes with contrasting spatial and temporal dynamics, and their results demonstrate that the performance of the data fusion methods is strongly associated with the spatial and temporal variance of the land cover [20]. Even though ESTARFM is better than STARFM in heterogeneous landscapes, it does not perform as well when predicting abrupt changes in land cover types. Additionally, the simulation accuracy is low when there is a large cloud cover. However, the difficulty in input data preparation (e.g., certain pairs of fine and coarse resolution images acquired on the same date) significantly limited their applicability. In order to accurately predict high-precision Landsat images and overcome the prediction error caused by large heterogeneous mutation regions, Zhu et al. proposed the flexible spatiotemporal data fusion model (FSDAF) and showed that FSDAF created more accurate fused images and retained more spatial detail than STARFM [21]. More importantly, FSDAF closely captures reflectance changes caused by land cover conversions, which is a major issue with the current spatiotemporal data fusion methods. In addition, FSDAF only requires a reference image for Landsat and MODIS and a MODIS image for the predicted time. FSDAF effectively reduces the amount of data input and is easily operated.

At present, global land cover and land surface phenology products (MOD09GA) are available at 500 m spatial resolution from MODIS [22,23], but the 500 m spatial resolution is too coarse for most crop fields. This resolution often results in mixed pixels of different vegetation or crop types, which may have very different phenological growth cycles [24,25]. The time-series MOD13Q1 250 m MODIS NDVI data are the primary source for most crop yield estimation models [26,27]. This study evaluated Landsat images of different dates to determine the optimal reference date for input fine image and then generated fused images of both MOD09GA and of MOD13Q1 and compared the results. In addition, we analyzed the effect of mixed pixels by comparing the accuracy of yield estimation of fusion products from the two different MODIS resolutions. 

Here, we extend the application of fusion images to field-scale study. The main objective of this study was to evaluate the ability of a Landsat–MODIS data fusion methodology to generate field scale data at 30 m resolution. Since crop yield can be predicted accurately using NDVI (and other indices) obtained at the peak of the growing season (peak greenness) [28,29,30], MOD13Q1 is a 16-day product, for which we can identify the specific DOY 230 because the day with the highest NDVI value is within the 16-day period for the MOD13Q1 product. In this way, we evaluated the fusion outputs by first comparing them to Landsat NDVI at DOY 230, and then, for the best outputs, by assessing their ability to predict yield. Specifically, our aims were to: (1) evaluate the fusion results of Landsat–MODIS data (MOD09GA and MOD13Q1); (2) evaluate the effects of different Landsat image dates on the results of the time-series fusion NDVI; and (3) assess the fusion results of Landsat–MODIS data (MOD09GA or MOD13Q1) over three cotton plots through MDI and review the impact on the accuracy of fused Landsat–MODIS images.

## 2. Materials and Methods

### 2.1. Study Area

**Figure 1:**
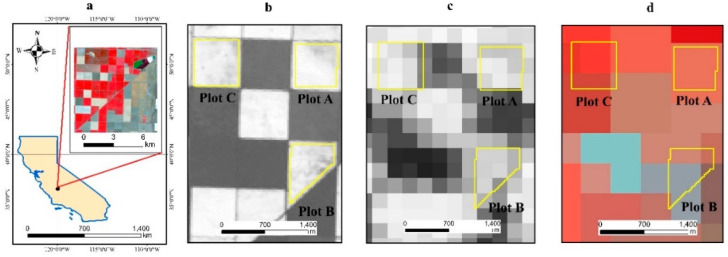
Map of the study area; (**a**) California map and Landsat 30 m image of the study area on DOY 206; (**b**) study plots overlain on Landsat NDVI on DOY 206; (**c**) study plots overlain on MOD13Q1 250 m (MODIS NDVI) image on DOY 193; (**d**) study plots overlain on MOD09GA 500 m image (Band 2, band 1, band 4) on DOY 206.

**Figure 2:**
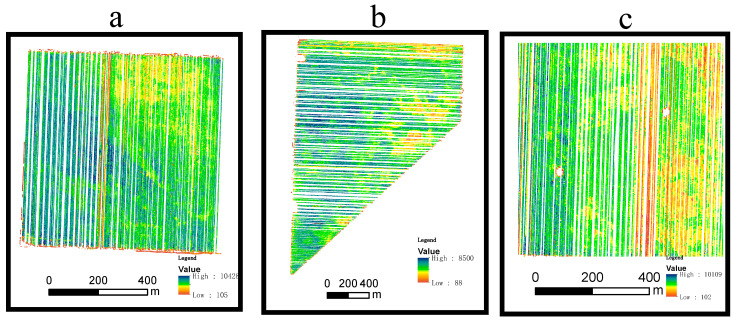
Cotton yield map (2002) of the study plots (**a**) plot A; (**b**) plot B; (**c**) plot C.

The study area is located on the western side of the southern San Joaquin Valley of California, USA, and has been extensively used for RS time-series research [31,32]. Cloud-free Landsat–MODIS pairs were available throughout the year because the San Joaquin Valley has a Mediterranean climate, with hot and dry summers (maximum of 40 °C and a day/night difference of approximately 16 °C) and cool and wet winters (average annual rainfall of 854 mm). The rainy season normally runs from November to April, and there was little rainfall recorded during the study period of May–September 2002 (https://www.usclimatedata.com/climate/california/united-states (accessed on 29 July 2021)). Three cotton plots (labeled A, B, and) were selected as the study area (Figure 1) because detailed cotton yield data for these fields were available for validation. Compared to MODIS images, Landsat NDVI has a relatively fine spatial resolution of 30 m (Figure 1b), and the mixed pixel problem is therefore not serious. Figure 1b–d present the Landsat and MODIS images of the three plots, each of which covers an area of approximately 9 pixels at the 250 m resolution of MOD13Q1 (Figure 1c) and only 1 pixel at the 500 m resolution of MOD09GA (Figure 1d). Figure 2 shows the yield map of the study plots.

### 2.2. Satellite Images

We have adopted an identifying nomenclature as follows: NDVI_L30_174_ identifies Landsat NDVI at 30 m resolution on DOY 174, NDVI_M250_ identifies NDVI from MODIS 250 m imagery, NDVI_F250_174_ identifies NDVI on DOY 174 from MODIS 250 m imagery fused with a Landsat image, and NDVI_F500_174_ identifies NDVI on DOY 174 from MODIS 500 m imagery fused with a Landsat image. Other products use the same format.

Landsat images (TM 5 and ETM 7) of the study area covering the cotton growth period from sowing in May to October were obtained for 2002 (http://earthexplorer.usgs.gov (accessed on 29 July 2021)). The temporal resolution of Landsat images was 8 days, and the spatial resolution was 30 m. Radiometric calibration and atmospheric correction were conducted during the preprocessing of the Landsat images. The Landsat NDVI (NDVI_L30_) time-series covering the May–September period (defoliant spraying in September) was calculated using the ‘Band math’ function of ENVI 5.1 (Esri, Redlands, CA, USA).

MOD13Q1 and MOD09GA products from 2002 were obtained from the NASA Reverb website (http://reverb.echo.nasa.gov (accessed on 29 July 2021)). The MOD13Q1 product includes 250 m NDVI data (NDVI_M250_) and quality assessment (QA) information, while the MOD09GA NDVI data (NDVI_M500_) was calculated from atmospherically corrected surface reflectance values. The MODIS Reprojection Tool (MRT; https://lpdaac.usgs.gov (accessed on 29 July 2021)) was used to convert HDF format images into ENVI standard format. In this study, to fit the requirements of the FSDAF data fusion model, MOD13Q1 and MOD09GA data were resampled to 240 m and 480 m in order to make better geo-spatial correction and integration with Landsat 30 m in integral proportion. Table 1 had show the dates and number of Landsat and MODIS images used in the study.

Note that MODIS and Landsat bands use different band number sequences in the FSDAF model. It can relate them using the following Landsat product bands 1, 2, 3, 4, 5, 7 vs. MOD09GA product bands 3, 4, 1, 2, 6, 7.

### 2.3. Yield Data

Yield data were collected using a cotton yield monitor onboard the harvester (ModelAG700, AGRIplan, Stow, MA, USA; www.agriplaninc.com (accessed on 29 July 2021)), and the yield accuracy (validated with manually harvested field plots) was estimated to be between 95 and 98% for the pixels of approximately 4.5 m by 4.5 m [33]. The cotton yield monitor uses an optical sensor to detect the volume of cotton as it passes through the machine. The crop volume is recorded on a storage device with location data from an onboard GPS, and a shape-file yield map is later produced using proprietary software. 

The yield map was produced in 3 steps [34]: 

Step 1: ASCII text and database files were converted into vector shape files using ArcGIS;

Step 2: The shape files were converted to raster, with the output raster cell size set to 0.5 m × 0.5 m to generate the yield map;

Step 3: The outlying yield values caused by grain time lag and yield surges were removed using a statistical identifier based on a moving average mean and standard deviation. If the yield was less than or greater than three standard deviations from the average, it was identified as an outlier and removed. 

### 2.4. FSDAF Model

The FSDAF model synthesizes frequent high spatial resolution images by blending frequent coarse spatial resolution data, such as those from MODIS, with less frequent high spatial resolution data, such as those from Landsat [21]. FSDAF requires only Landsat and MODIS images at the reference time and MODIS images at each of the predicted times, and therefore effectively minimizes the number of input images. By using a single reference image and implementing the model for each additional MODIS image over the growing season, a time-series of high-resolution NDVI images is generated. As FSDAF uses simple principles and requires only one fine-resolution image as an input, it has the potential to increase the availability of high-resolution time-series data in support of studies of rapid land surface dynamics. 

Implementation of the FSDAF model required six steps:

(1) manual delineation of the study area on the Landsat image at t1;

(2) calculation of the change in MODIS NDVI between t1 and t2;

(3) prediction of the Landsat NDVI image at t2 using fine-resolution temporal change in MODIS NDVI and calculation of residuals at each MODIS pixel;

(4) prediction of the Landsat NDVI image from the MODIS NDVI image at t2 with a Thin Plate Spline (TPS) interpolator [35], which is a spatial interpolation technique for point data based on spatial dependence;

(5) distribution of the residuals based on TPS prediction;

(6) obtaining the final prediction of NDVI at each Landsat pixel using information in the neighborhood.

**Figure 3:**
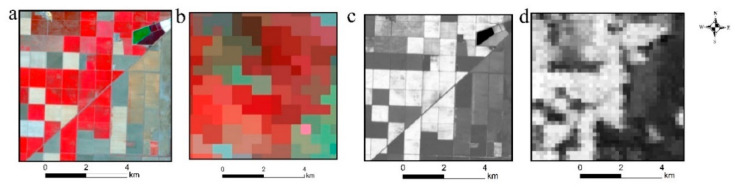
FSDAF input images (**a**) Landsat 30 m image on DOY 206; (**b**) MOD09GA 500 m image on DOY 206; (**c**) Landsat 30 m NDVI on DOY 206; (**d**) MODIS 250 m NDVI on DOY 193).

In this study, we compared the accuracy of the FSDAF fusion results using different MODIS products (MOD09GA and MOD13Q1) and Landsat images of different reference dates. Examples of DOY 206 imagery available for fusion are shown in Figure 3. 

### 2.5. Selection of Images for the Fusion Model

**Figure 4:**
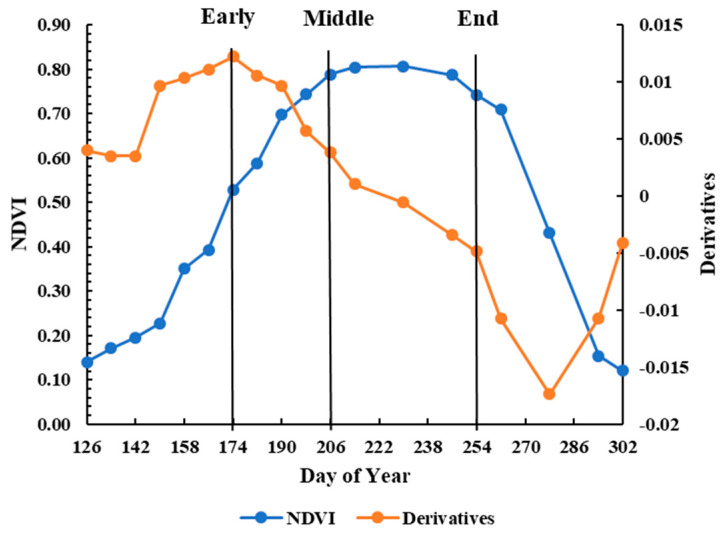
Time-series Landsat NDVI and derivatives curves over the cotton growing season.

Figure 4 shows the time-series NDVI_L30_ curve of cotton growth in the study area and the NDVI_L30_ derivatives obtained from the Landsat images from May to October in 2002 extracted from OriginPro 8.5 (https://www.originlab.com (accessed on 29 July 2021)). From the trend of NDVI derivatives, it is apparent that the cotton began to grow quickly on DOY 174, grew at a steady rate until DOY 206, and then fell gradually until DOY 254. We identified three stages, Early, Middle, and End, and selected DOY 230 as the peak of the growing season. A single Landsat image from each stage (DOY 174, 206, and 254, respectively) was selected as the reference date for FSDAF in order to compare the effect of the different dates on the accuracy of the fusion model in duplicating Landsat NDVI on DOY 230.

### 2.6. Mixed Degree Index (MDI)

**Figure 5:**
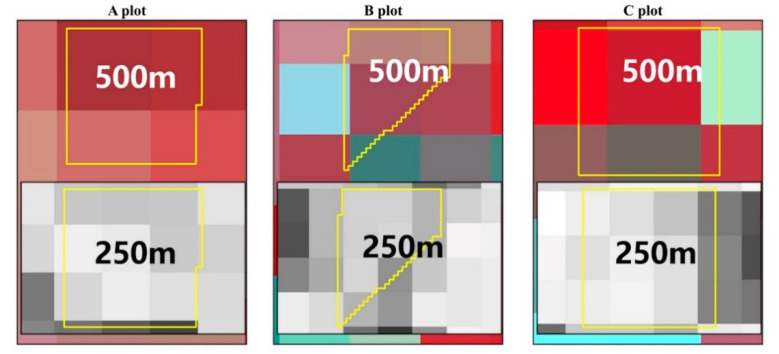
Three plots shown in MOD09GA (500 m) and MOD13Q1 (250 m).

**Figure 6:**
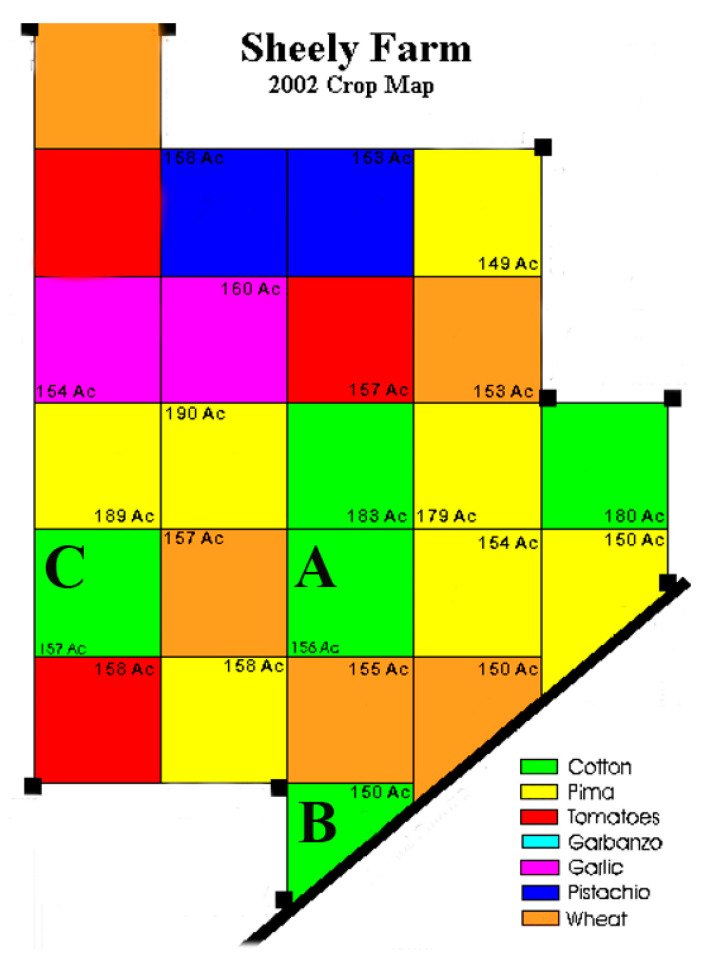
Three plots shown on the Sheely Farm crop map.

The MODIS products input into FSDAF model in this paper were MOD09GA and MOD13Q1, and the fusion process must account for the differences in vegetation change in the MODIS images. For example, the three different plots occupy different numbers of pixels; taking MOD09GA products as an example (Figure 5), plot A has four pixels, plot B has five pixels, and plot C has six pixels. From Figure 1, due to the serious mixed problem in the MODIS product, the surrounding area around the three plots is so different that it is important to consider the effects of crop structure and surrounding land cover on the fusion results (Figure 6). However, the pixels occupied by the plots are not all pure pixels; thus, it is necessary to analyze the mixing degree of the pixels occupied by the plots. In this paper, we first propose the MDI to evaluate the degree of pixel mixing and the fusion results of different plots; its calculation principles are described by Equations (1)–(3). It was constructed to evaluate the accuracy and time-series fusion results of the different cotton plots, after which the different yield estimation models were compared.

(1) Mixed pixel area ratio (M_PAR_)

(1) $$M_{\mathrm{PAR}}=\frac{S_{P}}{S_{W}}*\frac{S_{P}}{S_{Y}}$$

Note: *S_P_* is the area of the pixel of the plot; *S_w_* is the area of a pure pixel (250 m × 250 m or 500 m × 500 m); *S_Y_* is the area of the plot.

(2) NDVI proportion (NDVI_Pro_)

Different proportions of NDVI values of surrounding ground features are different in the two MODIS products (250 m or 500 m); this paper uses the NDVI_Pro_ to consider the pixel NDVI influenced by MODIS products of different resolutions.

(2) $${\mathrm{NDVI}}_{\mathrm{Pro}}=\frac{ABS({\mathrm{NDVI}}_{M}-{\mathrm{NDVI}}_{L})}{MAX\left({\mathrm{NDVI}}_{L},{\;\mathrm{NDVI}}_{M}\right)}$$

Note: NDVIM is the NDVI of the MODIS product (250 m or 500 m); NDVI_L_ is the NDVI of Landsat (30 m).

(3) Mixed degree index (MDI)

(3) $$\mathrm{MDI}=\sum_{i=1}^{n}M_{\mathrm{PAR}}*{\mathrm{NDVI}}_{\mathrm{Pro}}$$

Note: (a) Number the pixels one by one, from left to right, top to bottom, (1, 2, 3... *n*); (b) Pixels for which the degree is less than 1% are not included; (c) The lower right side of plot B is largely affected by fallow farmland, and it is divided into different farms by a road, which covers 13% of the total area (Figure 5 and Figure 6); (d) MDI values from 0 to 1.

### 2.7. Yield Estimation Model

Correlation regression analysis was used to analyze the correlation between the yield and the NDVI_L_ or the predicted NDVI from the fusion results (NDVI_F_). The regression model was established with SPSS19.0 (http://www.ibm.com (accessed on 29 July 2021)). Therefore, this paper took the NDVI_L_ and the NDVI_F_ as the input variables (both NDVI_F500_ from MOD09GA and NDVI_F250_ from MOD13Q1) for yield estimation and analyzed the accuracy to validate the feasibility of fusion images for yield estimations.

Yield Model(*y*) = a∗*x* + b (4)

where *y* is the predicted yield, *x* is NDVI, predicted NDVI from fusion result, respectively; a and b are coefficients.

### 2.8. Accuracy Evaluation Method

(1) Evaluation of FSDAF 

The model was evaluated using the adjusted decision coefficient (*R*^2^_Adj_, Equation (5). From the scatter between the NDVI_F_ and the NDVI_L_, the *R*^2^_Adj_ was calculated, and the fusion results were evaluated from *R*^2^_Adj_.

(5) $$R_{\mathrm{Adj}}^{2}=\frac{\sum_{i=1}^{n}{\left(x_{i}-\bar{x}\right)}^{2}}{\sum_{i=1}^{n}{\left(x-\bar{x}\right)}^{2}}$$

where x is the NDVI_F_, $x_{i}$ is the NDVI_L_, and $\bar{x}$ is the average NDVI_L_.

The closer to 1 the *R*^2^_Adj_, the better the FSDAF fusion result.

(2) Evaluation method for the yield estimation model

The general yield model equation is shown as Equation (4). The yield models were evaluated using the decision coefficient (*R*^2^) and the root mean square error (RMSE) [36,37].

### 2.9. Flow Chart of Data Analysis, Model Validation, and Evaluation

**Figure 7:**
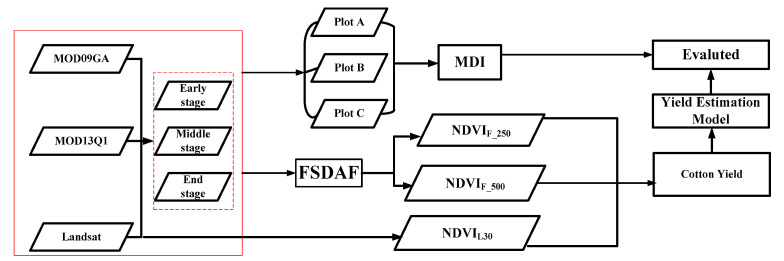
Flow chart of the technique employed in this study.

In this paper, MOD09GA and MOD13Q1 products and Landsat images were input into the FSDAF fusion model, and NDVI_F_250_ and NDVI NDVI_F_500_ were obtained. In addition, MDI was constructed to evaluate the mixing degree of different plots in the different MODIS products. Finally, the cotton yield estimation models were established, and the accuracy evaluation was carried out. A flow chart is shown in Figure 7. 

## 3. Results

### 3.1. Time-Series NDVI of the MODIS and Landsat Images

**Figure 8:**
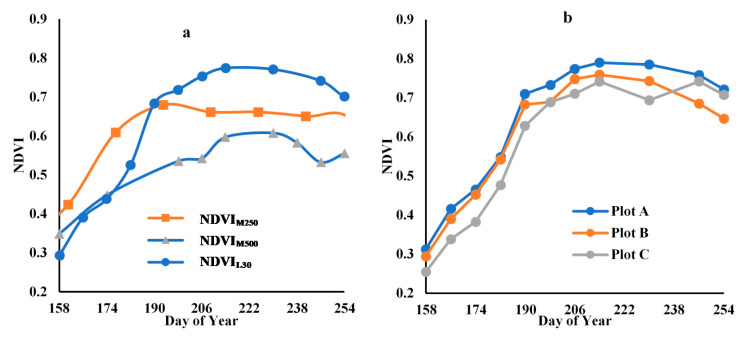
Time-series NDVI curve (**a**) NDVI_M500_, NDVI_M250_, and NDVI_L30_; (**b**) NDVI_L30_ of the three cotton plots).

Figure 8 shows linear interpolations of NDVI across the time-series of each of the input images for all three plots. Landsat (NDVI_L30_) and MODIS images (especially NDVI_M250_) show a similar time trend, even though the spatial resolution of the two MODIS products is much greater, at 250 m and 500 m, than the 30 m of the Landsat images. The problems caused by mixed pixels, as outlined by Fitzgerald et al., may be masked by the fact that the mix of vegetation within the large pixels in this agricultural setting relates to different crops that are all at relatively the same growth stage [38]. The time-series NDVI_L30_ and NDVI_M250_ show some difference in the early portion of the cotton growth season (Figure 8a), which align almost perfectly by the middle of the season and then diverge again in the latter portion. The NDVI_M500_ series has a similar trajectory to NDVI_L30_, but the values are somewhat lower than those of NDVI_L30_ and NDVI_M250_, especially in the middle portion of the cotton growing season, where there is an unexplained dip in reflectance. 

Figure 8b shows the time-series NDVI_L30_ of the three cotton plots of the study area. The three plots have similar growth curves, with plot A exhibiting slightly better growth than plots C and B. From this similarity in growth pattern, it can be assumed that any plot difference in yield estimation accuracy by NDVI_F250_ and NDVI_F500_ is due to the difference in pixel mixing rather than crop condition.

### 3.2. MDI of A/B/C at Different Spatial Resolutions 

**Figure 9:**
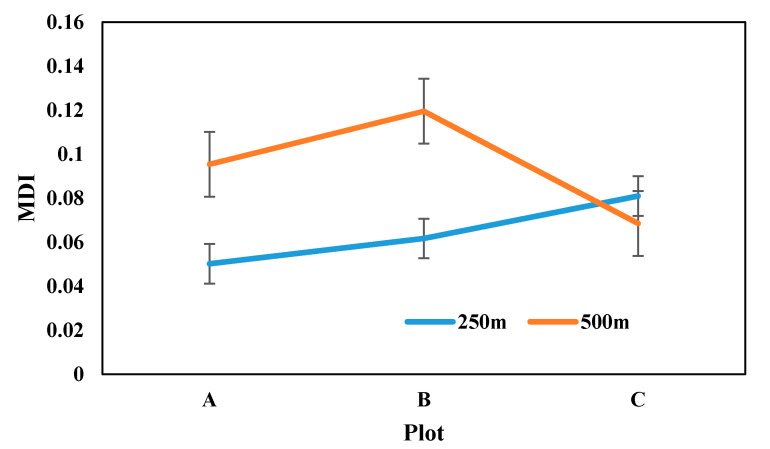
MDI curves of the three plots at the scales of 250 m and 500 m.

Figure 9 shows the MDI of the three plots at 250 m and 500 m spatial resolution. It can be seen from Figure 9 that the MDI in the 500 m MODIS product of plot A is better than that of B but slightly lower than that of C. Figure 5 shows that the proportion of pure pixels at 250 m occupied by plot C was greater than that of A. For plot B, the MDI in 250 m product was higher than that in A and C. With respect to the location of plot B, the MDI was increased due to the influence of the plot shape and surrounding crop types (Figure 6). B was largely affected by fallow farmland, and it was divided into different sections by a road, which covered 13% of the total area. 

### 3.3. Landsat–MODIS Fusion Results

#### 3.3.1. Results of the Fusion of Landsat at Different Reference Dates with 500 m MOD09GA

**Figure 10:**
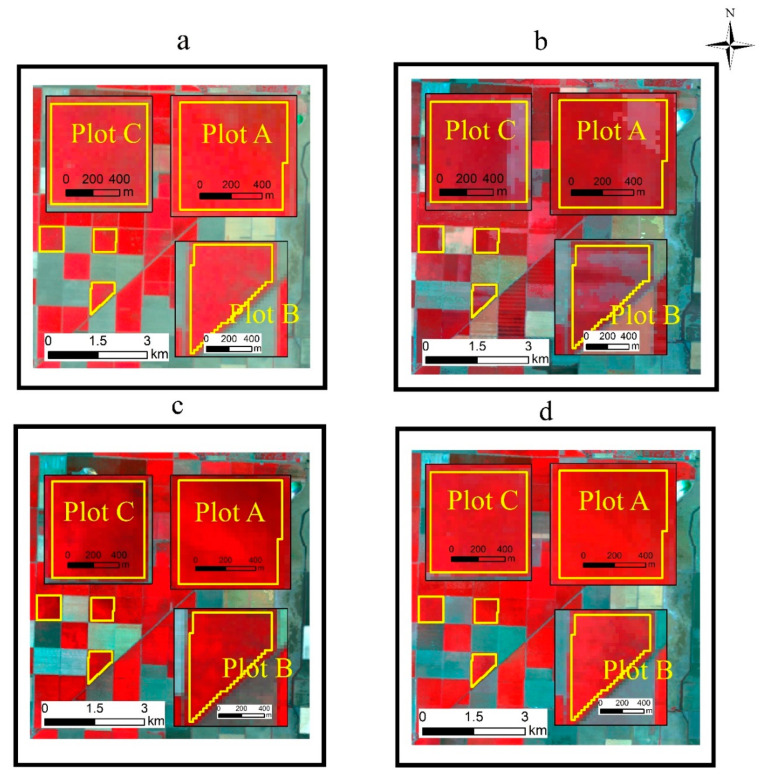
A visual comparison of fusion results; (**a**) Landsat image on DOY 230; (**b**–**d**) DOY 230 image from the fusion of reference Landsat images of DOY 174, DOY 206, and DOY 254 with MODIS 500 m imagery.

Three single-date Landsat NDVI images (DOY 174, DOY 206, DOY 254) were used in FSDAF to explore the influence of different reference dates on the accuracy of predicting the peak of the growing season through Landsat/MODIS fusion (NDVI_F500_). As an example of the results, Figure 10 provides a visual comparison of fusion results at peak season (DOY 230) using Landsat images on DOY 174, DOY 206, and DOY 254 as reference images. The overall view of the fusion results is similar to the Landsat image, even capturing differences in crop conditions, observable as lighter and darker areas within the images.

**Figure 11:**
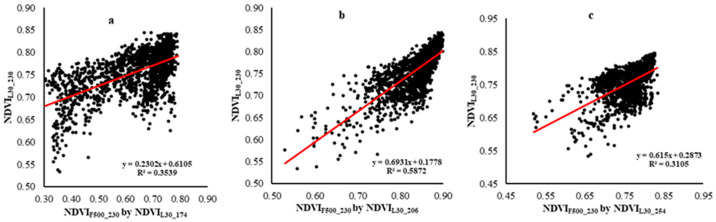
Scatterplots showing the relationship between the fusion products NDVI_F500_ and NDVI_L30_; NDVI_L30_230_ versus NDVI_F500_230_ using (**a**) NDVI_L30_174_, (**b**) NDVI_L30_206_, and (**c**) NDVI_L30_254_ for all pixels of 3 plots).

The NDVI values of the fused 500 m MODIS (NDVI_F500_230_) with three different reference Landsat images over all three plots were compared to Landsat NDVI at peak greenness (NDVI_L30_230_) using scatterplots and *R*^2^ values. Table 2 provides the results of linear regression using reference images from the early, middle, and end stages of the growing season. A comparison of *R*^2^ values shown in Figure 11 and Table 2 indicates that NDVI_F500_230_, which uses NDVI_L30_206_ as a reference image, has a higher correlation with Landsat at DOY 230 than fused images using NDVI_L30_174_ or NDVI_L30_254_ as reference images. These results indicate that using a reference image from the middle stage of the crop growth season provides a fusion product that better represents peak growth than using reference images from early or late in the growing season.

#### 3.3.2. Results of the Fusion of Landsat at Different Reference Dates with 250 m MOD13Q1

**Figure 12:**
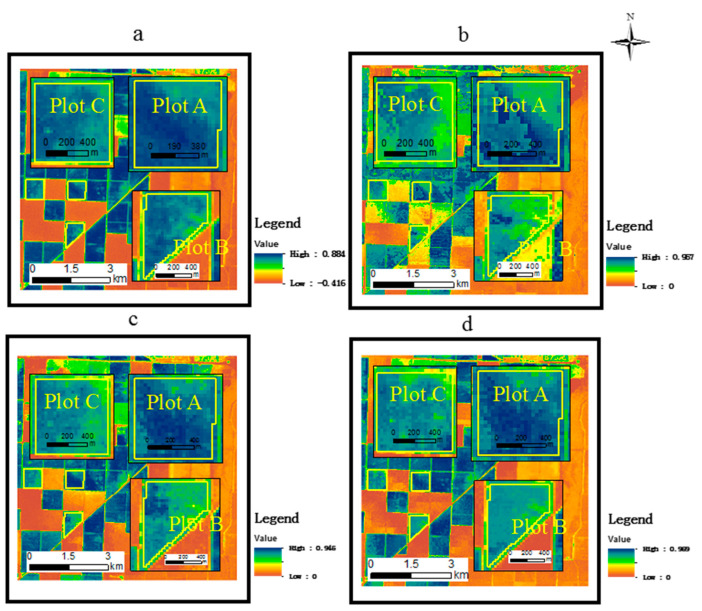
A visual comparison of fusion results; (**a**) Landsat NDVI image on DOY 230; (**b**–**d**) image on DOY 225 from fusion of MODIS 250 m imagery (NDVI_F250_225_) with reference Landsat images on DOY 174, DOY 206, and DOY 254.

Landsat images from the early, middle, and end stages of the cotton growth season were used in FSDAF with NDVI_M250_, as was completed with the 500 m MOD09GA product. Figure 12 presents a visual comparison of Landsat at peak growth (NDVI_L30_230_) with fused 250 m MODIS images (NDVI_F250_225_) using three different Landsat reference images (DOY 174, DOY 206, and DOY 254). 

**Figure 13:**
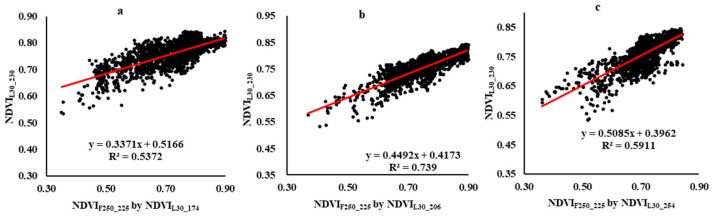
Scatter plots of NDVI_F250_225_ using different reference dates versus NDVI_L30_230_. NDVI_L30_230_ versus NDVI_F250_225_ using (**a**) NDVI_L30_174_, (**b**) NDVI_L30_206_, and (**c**) NDVI_L30_254_ for all pixels of 3 plots).

Figure 13 shows scatterplots of NDVI_L30_230_ versus NDVI_F250_225_ using different reference dates. These scatterplots show slightly higher correlation values than those of MOD09GA, but the trend is the same, with NDVI_F250_225_ using NDVI_L30_206_ as reference being slightly better than those using NDVI_L30_174_ or NDVI_L30_254_. Table 3 presents results for each plot separately and shows an anomaly for plot B, in which the results using both early and late-season reference images provide higher R^2^ values than the mid-season reference image. This may be due to the unexplained flattening of the growth curve around DOY 200 in plot B as revealed in Figure 8b, or it may be due to the irregular shape of plot B compared to the square outlines of plots A and C. This irregular shape may exacerbate the influence of mixed pixels at the peak of the growing season. It can be observed in Figure 12 that plot B has a more uniform pattern that closely matches the Landsat image when fused with Landsat from DOY 254. 

#### 3.3.3. Analysis of Time-Series Fusion Results

**Figure 14:**
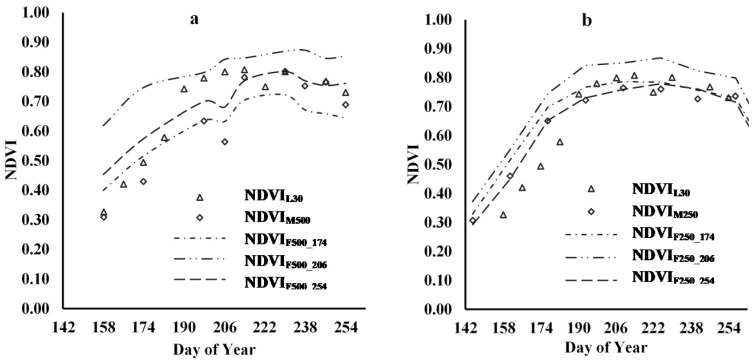
Time-series data of NDVI from Landsat, MODIS, and different fusion products for plot A (**a**) 500 m MOD09GA; (**b**) 250 m MOD13Q1.

Figure 14 shows time-series curves of NDVI of Landsat, MODIS, and various fusion products. Figure 14a shows the time-series NDVI_F500_ using different reference Landsat images of plot A as an example, while Figure 14b shows the same information for NDVI_F250_. The time-series NDVI_F250_ values display less variation and are more closely aligned with the Landsat series than the 500 m images, especially in the middle and end stages of the growing season. 

Although the farm containing the study area is large, each cotton plot in our study intersected with only four to six 500 m MOD09GA pixels, and it appears that the mixture of cover types within these pixels caused variability and divergence from the time-series NDVI of Landsat. For the 250 m MOD13Q1 fusion products, the smaller pixel size appears to avoid the mixing problem to a certain extent, thereby enhancing the accuracy of fusion products generated with MOD13Q1 imagery. There are mixed pixel problems associated with all MODIS products, but the coarser the resolution, the more serious the mixing problem [39].

**Figure 15:**
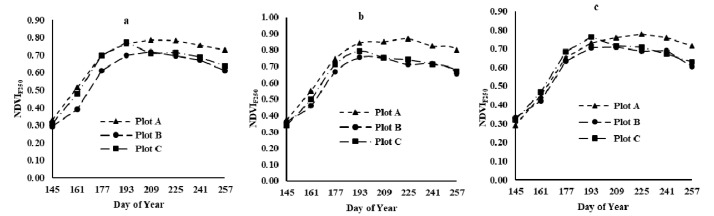
Time-series NDVI_F250_ fused with NDVI_L30_ of the three plots (**a**) fused with NDVI_L30_174_; (**b**) fused with NDVI_L30_206_; (**c**) fused with NDVI_L30_254_.

Based on the relationships displayed in Figure 11, which indicate that the results from fused NDVI_M250_ are more closely aligned with Landsat results, we selected the 250 m products for further detailed yield analysis. Figure 15 shows the time-series of NDVI_F250_ fused with NDVI_L30_174_, NDVI_L30_206,_ and NDVI_L30_254_ for the three study plots. Comparing Figure 15 with Figure 8a, it can be seen that the predicted fusion results are consistent with the actual Landsat time-series NDVI, with little difference among the three plots or different reference dates. 

### 3.4. Correlation Analysis between Cotton Yield and Time-Series Fusion NDVI

**Figure 16:**
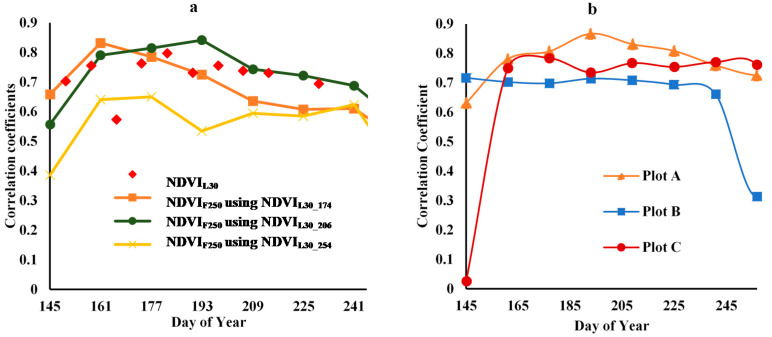
Correlation coefficients between NDVI_L30_, NDVI_F250_ fused with 3 dates of Landsat (DOY174, 206, 254) and cotton yield (**a**) correlation coefficient results from NDVI_F250_ for three plots; (**b**) correlation coefficient results from MOD13Q1 and Landsat NDVI_L30_206_ of three plots.

The correlation coefficients between time-series NDVI from different image products and cotton yield are shown in Figure 16. Figure 16a shows the correlation coefficients of NDVI_L30_ and NDVI_F250_ using three different fusion reference dates for all pixels in all three plots. The data show a strong correlation between NDVI_L_ and yield for all image dates, and a similarly close relationship (except early in the season) between yield and NDVI_F250_ using Landsat on DOY 206. Both NDVI_F250_ using Landsat on DOY 174 and NDVI_F250_ using Landsat on DOY 254 display considerably lower and divergent trends in correlation with yield. Figure 16b shows the correlation coefficients between cotton yield and NDVI_F250_ using Landsat NDVI_L30_206_ as a reference for each of the three plots. As shown in Table 3, this fusion product has high accuracy with respect to duplicating Landsat values at the peak of the season, and the result is a good correlation (above 0.6) with yield in all three plots. There are anomalies at the start of the season in plot C and at the end of the season in plot B, which may be explained by the fact that cotton growth during the start of the season in plot C was a little worse than plot A and B. However, plot B had poor growth at the end of the season. There is a strong correlation between cotton yield and both time-series NDVI_L30_ and NDVI_F250_, and the fusion results from DOY 206 as reference using ‘Middle Stage’ imagery are best for all three plots (Table 3).

**Figure 17:**
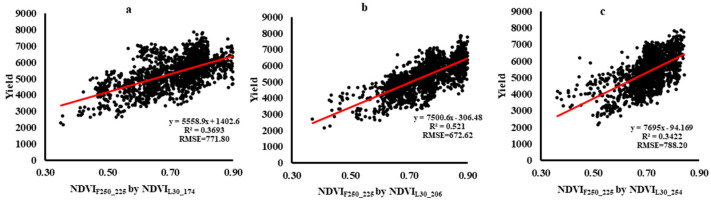
Scatter diagram between fusion result and cotton yield for three plots (**a**) NDVI_F250_225_ by NDVI_L30_174_; (**b**) NDVI_F250_225_ by NDVI_L30_206_; (**c**) NDVI_F250_225_ by NDVI_L30_254_.

The Scatter diagram between fusion result and cotton yield for three plots is shown in Figure 17. It shows the R^2^ and RMSE had a high accuracy on fusion results based on NDVI_F250_225_ by NDVI_L30_206_, with the RMSE 14.73% and 17.26% higher than that from NDVI_L30_174_ and NDVI_L30_254_. In addition, the time-series NDVI (Figure 14) and the correlation coefficients (Figure 16) indicate that when the prediction date (t2) is closer to the date of the input Landsat image for the fusion model (t1), the fusion results are more accurate. 

## 4. Discussion

In this study, we analyzed the impact of different reference dates on the ability of fusion results to represent the peak of the growing season. We selected MOD09GA and MOD13Q1 products to blend with Landsat data using the FSDAF model, and found that peak NDVI from the fusion of both MODIS products is influenced by the date of the Landsat images used as reference. Specifically, the fusion result for DOY 230 in the middle of the growing season using Landsat from DOY 206 as reference follows the Landsat NDVI pattern for DOY 230 with higher accuracy than the fusion products using Landsat from DOY 174 (early season) or DOY 254 (late season) as reference images. Compared to the study of Jamshidi et al. [40], it also takes into account the results of reference image fusion in different input periods for evapotranspiration. The optimal fusion results were in the early growing stage and harvest time, which is different from this study. We obtained better fusion accuracy on the middle stage of cotton growth because the crop grows well and the plots are uniform. Additionally, the soil, meteorological conditions, and environmental impact are small for remote sensing images and crop conditions. We also determined that the higher accuracy of a fused 250 m MODIS image can lead to a very high level of yield correlation (*R*^2^ = 0.83) than a fused 500 m MODIS image, indicating that the 250 m MOD13Q1 product with the higher spatial resolution is preferable for use in fusion models destined for field-level yield estimation. We might not achieve good accuracy with MOD09GA due to the negative effect of mixed pixels, as outlined in other studies [23,41]. With respect to the three plots, the 250 m fusion results are consistent, with the accuracy of fused images using a reference Landsat image from the middle of the season being highest, and accuracy for plot A being higher than for plot B or C. We attribute the higher accuracy for plot A to be due to the fact that the pixel boundaries align closely with the plot boundaries, resulting in more pure pixels. Additionally, the MDI also shows the same results that MDI plot A for 250 m is higher than for plot B or C. For 500 m, there are strong correlation between fusion results and MDI. However, MDI and fusion results are not relevant in fusion results of 250 m among plot B and C. The two plots have serious mixed pixel problem, but because of the irregular shape of B, resulting in a small number of mixed cells, resulting B < C(MDI). However, compared at the 30 m scale for fusion results, plot C obtained a better accuracy than B.

At this scale there can be problems associated with mixed pixels in all MODIS products, but it appears that the chance alignment of pixels with field boundaries can have a positive impact on accuracy. It is noteworthy that the accuracy of yield estimation in plot B using a late-season reference image (*R*^2^ = 0.77) is higher than for the mid-season reference image and only slightly lower than the highest accuracies (using mid-season imagery) in plots A and C. This result needs further study.

In this paper, not only the number of pixels and the proportion of distribution of two MODIS products but also NDVI_Pro_ was considered, and the MDI was constructed to analyze the fusion results. At the field scale, MDI is important for the fusion study because of the mixed pixel problems in MODIS products. Additionally, more fusion models and multi-source RS images should be tested to verify it. This study represents considerable progress in the evaluation of crops from regional land use to the field scale, and the factors affecting the application accuracy at the field scale include the differences in plot shape and surrounding crop type. In the future, the mixed pixel problem of MODIS products should be considered in the development of the fusion model at the field scale. 

The data fusion model considers all plots as homogeneous farmland. However, crop type must be considered for crop yield estimation, and the crop type of one plot can differ from that of another plot. Therefore, the data fusion model maintains the overall spatiotemporal crop and land cover patterns but cannot obtain adequate results in areas with different crop phenology or small plots. A yield estimation model for different crops and different crop phenology will be considered in the future. Although currently there are a number of higher resolution satellite sensors such as Sentinel-2 available [42], we restricted our study to older imagery due to the availability of time-series Landsat data and high-resolution yield data at this time at this site, but it is a benefit to temporal change studies of a methodology for generating historical high-resolution field-level yield estimates.

## 5. Conclusions

In this study, we extended the application of data fusion to the field scale to estimate crop yields. Additionally, we analyzed time-series Landsat and MODIS NDVI data and assessed the importance of different Landsat reference date for fusion results. First, this study found that the NDVI_F_ from the fusion data is significantly influenced by the dates of the Landsat images during the cotton growing season. Second, the fusion result and the yield estimation model from MOD13Q1 and Landsat TM5 are more accurate than those of MOD09GA and Landsat, which proves that the 250 m MOD13Q1 product with the higher spatial resolution is preferable for use in fusion models rather than the 500 m MOD09GA product. Third, yield estimation model accuracy was influenced by the MDI of the three plots, the number of pixels, and the proportion of distribution of two MODIS products. The NDVI_Pro_ can influence the fusion results at 250 m and 500 m spatial resolutions. This study estimates crop yields using fusion images from the MOD13Q1 product at the field scale; these images can be used for vegetation monitoring and yield prediction with RS at the field scale. 

Despite the success of fusion study at the field level, there are additional areas that need study; (1) in future research, Sentinel-2 and Landsat images could be combined to improve the spatial and temporal resolution and enable a more precise estimation of crop yield. (2) In addition to NDVI, the Enhanced Vegetation Index (EVI) could be an alternative for densely vegetated areas. EVI has been shown to provide a high degree of separation of vegetative reflection and could enhance yield estimation results. In our next study, we will test MODIS EVI in the FSDAF model and evaluate the accuracy. 

## Figures and Tables

**Table 1:** Dates and number of Landsat and MODIS images used in the study over the cotton growing season from May to October in 2002.

Product	Sensors	Date	Number of Images
Landsat	TM 5	6 May; 22 May; 7 June; 23 June; 1 July; 17 July; 2 August; 26 August; 11 September; 27 September; 13 October; 29 October;	12
	ETM 7	14 May; 30 May; 15 June; 9 July; 25 July; 18 August; 3 September; 19 September; 5 October; 21 October;	10
MODIS	MOD13Q1	25 May; 10 June; 26 June; 12 July; 28 July; 13 August; 29 August; 14 September;	8
	MOD09GA	7 June; 23 June; 17 July; 25 July; 2 August; 18 August; 26 August; 3 September; 11 September;	9

**Table 2:** Results of linear regression between the fusion product NDVI_F500_230_ using different reference dates and NDVI_L30_230_ in 3 plots.

Plot	Early Stage (NDVI_L30_174_)	Middle Stage (NDVI_L30_206_)	End Stage (NDVI_L30_254_)
A	*y* = 0.3225*x* + 0.5601 *R*² = 0.3298	*y* = 1.5028*x* − 0.5154 *R*² = 0.6586	*y* = 0.8208*x* + 0.1313 *R*² = 0.1401
B	*y* = 0.2752*x* + 0.5978 *R*² = 0.3775	*y* = 0.6046*x* + 0.2604 *R*² = 0.652	*y* = 0.5426*x* + 0.3506 *R*² = 0.2626
C	*y* = 0.2282*x* + 0.5908 *R*² = 0.3962	*y* = 0.8174*x* + 0.0569 *R*² = 0.6599	*y* = 1.092*x* − 0.0946 *R*² = 0.4449
ABC	*y* = 0.2302*x* + 0.6105 *R*² = 0.3539	*y* = 0.6931*x* + 0.1778 *R*² = 0.5872	*y* = 0.615*x* + 0.2873 *R*² = 0.3105

**Table 3:** Results of linear regression between the fusion product NDVI_F250_225_ using different reference dates and NDVI_L30_230_ in three plots.

Plot	Early Stage	Middle Stage	End Stage
A	*y* = 0.3669*x* + 0.5008 R² = 0.4933	*y* = 0.6458*x* + 0.2429 R² = 0.831	*y* = 0.6934*x* + 0.253 R² = 0.6195
B	*y* = 0.3911*x* + 0.4802 *R*² = 0.6298	*y* = 0.4805*x* + 0.4025 *R*² = 0.6082	*y* = 0.9684*x* + 0.0901 *R*² = 0.7703
C	*y* = 0.224*x* + 0.5886 *R*² = 0.3148	*y* = 0.4911*x* + 0.389 *R*² = 0.800	*y* = 0.3436*x* + 0.5077 *R*² = 0.4459
ABC	*y* = 0.3371*x* + 0.5166 *R*² = 0.5372	*y* = 0.4492*x* + 0.4173 *R*² = 0.739	*y* = 0.5085*x* + 0.3962 *R*² = 0.5911
